# Perspectives on Spare Embryos amongst IVF users: An Exploratory Study from a Selected District of the Southern Indian State of Karnataka

**DOI:** 10.1007/s11673-024-10359-7

**Published:** 2024-07-29

**Authors:** Salik Ansari, Ravi Vaswani

**Affiliations:** 1 Centre for Ethics, Yenepoya (Deemed to be University), Mangalore, India; 2 Sangath, Bhopal, India; 3 Department of Internal Medicine, Yenepoya (Deemed to be University), Mangalore, India; 4 Centre for Ethics, Yenepoya (Deemed to be University), Mangalore, India

**Keywords:** IVF, Reproductive ethics, Spare embryos, Assisted reproductive technologies

## Abstract

Perspectives of IVF users on their spare embryos is a less explored subject in the Indian context despite the country’s population and abundance of IVF clinics. We conducted a qualitative study using in-depth interviews in a selected district of the Indian state of Karnataka. Seven individuals were recruited independently of any assistance from an IVF clinic. The interviews explored participants’ knowledge and perception of the spare embryos using a set of guiding questions exploring the theme of the informed consent process, views on research, preferences for embryo donation, the role of family and the dynamics of decision-making, amongst other things. The interviews were qualitatively analysed using Corbin and Strauss’s grounded theory approach. Our findings reveal that the participants do not learn about the prospects of spare embryos from the very start of their IVF journeys, and they may not be informed about the various options available to decide the fate of the spare embryos. Irrespective of their views on research and moral perceptions of embryos, participants expressed a sense of responsibility and ownership towards their embryos and a general reluctance to donate them. Our findings have implications for guiding future inquiries on this subject, which can better the informed consent process and unravel the role of ownership in the ethics of spare embryos in the Indian context.

## Introduction

In vitro Fertilization (IVF) is one of the most common forms of Assisted Reproductive Technology used in the treatment of infertility in humans by fertilizing oocytes (eggs) using sperm in the controlled conditions of a laboratory (Seidel 2015). Since the implantation of the fertilized oocytes (embryos) into the uterus is not guaranteed in an IVF cycle, a large number of embryos are created and cryopreserved to allow for multiple numbers of embryos to be transferred (Committee on Pediatric Research and Committee on Bioethics 2001). The production of excess embryos, therefore, improves the success rate as well as minimizes the cost and the risk associated with multiple attempts at hyper-stimulation of ovaries.

Post successful IVF procedures there may be surplus embryos that are no longer needed or desired by the couple. Such embryos are referred to as “spare embryos” (also, supernumerary or leftover embryos) (Mehta 2014). When the couple is undergoing the process of IVF these embryos are stored solely for the sake of the couple, however, when the couple has completed (or quit) the IVF procedures the fate of these “spare” embryos is uncertain.

In theory, a spare embryo could have the following future options:

- It can be donated to another couple who is undergoing IVF and needs it;
- It may be donated for research of any kind;
- It may be donated to the IVF centre itself for training purposes. Trainee lab workers may require training on techniques like preimplantation genetic diagnosis (PGD) and embryo handling (Boys and Walsh 2017);
- It may be abandoned i.e.; the couple has intentionally left the embryo at the ART centre without taking any decision on its fate and is non-responsive to the clinic’s request regarding the same;
- It may be stored voluntarily (up to infinity);
- It may be discarded/destroyed.

The execution of each of these options raises its distinct set of ethical issues and commissioning couples may experience emotional difficulty and hesitation when it comes to deciding on the fate of spare embryos (De Lacey 2005; McMahon et al. 2003). Both knowledge and perception of IVF users on their spare embryos could play a vital role here. The amount of information the couple is provided via the informed consent process (ICP) or counselling, on the various available options along with their conceptualization of embryos, can serve as a crucial factor in influencing their decisions (McMahon et al. 2003) along with their own views and beliefs on embryos. This has prompted studies in various parts of the world to explore the perspectives of IVF users on their spare embryos. Such studies have the potential to inform perspectives on embryo donation, embryo freezing, creation of embryos solely for research, discernment of the spare embryos, creation of any surplus embryos (via hyperstimulation), and technologies like IVF in general. However, a great majority of these studies are based in high income countries only.

It is in this context that we conducted a qualitative study exploring the perspectives on spare embryos amongst IVF users of Dakshina Kannada (DK), a district in the southern Indian state of Karnataka. The findings of our study adds to the empirical case of Indian IVF users on the discourse surrounding spare embryos. Such inquiries are essential to inform policies, strategies for counselling IVF patients and strategies for embryo storage and donation.

## Methodology

We used a qualitative approach as the evolving and flexible nature of a qualitative approach helps explore the inner experiences of the participants and how they form and transform meanings with regard to the embryos.

Study site: Our study was conducted in Dakshina Kannada (South Canara), a southern coastal district of the Indian state of Karnataka. The total population of the region is 20,89,649, with 88.57 per cent literacy as per the 2011 census (Dakshina Kannada District Administration 2024).

Inclusion criterion: Residents couples of Dakshina Kannada (DK) who underwent IVF procedures at any point in their life. Factors like the location of the IVF centre, the outcome of the IVF procedure, the involvement of a donor gamete, and the age of the participants, amongst others, did not affect the participant’s inclusion.

Exclusion criterion: Couples still undergoing IVF treatment, i.e. those who are currently in some stage of an IVF cycle. This is because people on the IVF journey are likely too preoccupied with the outcome. Also, this population subgroup would be harder to recruit unless one collaborates with IVF clinics.

Recruitment: The process of recruitment consisted of three stages—identifying the potential participants, approaching them, and obtaining consent.

Identifying the potential participants: Convenient sampling and snowballing were the methods used to identify and recruit potential participants. We asked health professionals, IVF providers, laypersons like auto-rickshaw drivers, students, gym instructors, and shopkeepers whether they knew someone in a personal capacity who meets the inclusion criterion of our study. If they did, then flyers/participant information sheets (in English and/or local language) were handed to them to forward to the potential participant.

Approaching the participants: Only if and when the potential participants were willing to be contacted by our research team did the Principal Investigator (PI) contact the participant. Our study design allowed the participants to choose the date, time, and mode (telephone, video call, or in-person) of the interview.

Obtaining consent: Written informed consent was obtained from all participants. For participants who did not understand English, we developed the informed consent document in the local language with the help of a professional translator. Consent for audio recording was obtained separately.

Study tool: We conducted interviews with the help of a set of guiding questions and a demographic sheet. We developed open-ended questions on certain domains of inquiry using the existing literature on this subject—the informed consent process, views on embryos, preferences regarding embryos, concerns during the process of IVF, decision-making process, knowledge and views on research using embryos, amongst others. More domains emerged as interviews were recorded- seeing the embryos, prior research on IVF, embryo adoption, relationship with the IVF provider, and eugenics, amongst others. To minimize response bias, the questions designed were checked for inherent bias by reviewing them several times. We ensured that the question did not lead the respondent to a definite response.

The demographic sheet captured relevant information like- the number of IVF cycles, religious adherence, education, employment status, and use of donor gametes. It was indicated clearly on the sheet, as well as informed orally, that answering questions is not mandatory.

Interview: The language of the interview was either English or Kannada, depending upon the participant’s preference. In the case of Kannada, a bilingual interpreter would accompany the PI during the interview with the participant’s permission. One interview was obtained per couple, and they were given full authority to decide whether they wanted to be interviewed together, separately, or only one person wanted to be interviewed. Post-interview, the participants were requested to suggest other participants for the study (snowballing). Participants had the full right to discontinue and/or withdraw their participation from the study at any point.

All the interviews were transcribed in English from the audio recordings. For interviews in the local language, the interpreter would translate the participant’s words into English during the conduct of the interview. All interview transcripts were anonymized and only the research team had access to identifiable information.

Analysis: We used the grounded theory (GT) approach for the analysis of data. GT allows us to look at the data from various angles and to come up with comprehensive explanations. It is useful to explore beliefs, meaning, rationale, logic, emotion, response to the problem, and action interactions among others. Besides, these steps of GT have been proven to be culturally sensitive and the theory can always be revised and updated as more information comes in (Corbin and Strauss 2014).

The following steps were involved in the analysis-

(i) Open coding: The interview transcript was read line by line. All transcripts were reviewed, and the data was broken into smaller parts to examine, compare for similarities and differences, and conceptualize open codes.

Open codes are the words used by the PI to denote the interpreted meaning of the data (Corbin and Strauss 2014). If a statement could mean more than one thing, it was coded into more than once generating more than one open code for a similar statement. Memos were also written in this process. Memos are the questions, thoughts, and inputs which come into the researcher’s mind while doing the process of coding. Memos subsequently help to relate the codes with each other.

The free software WEFT QDA was used to perform open coding. We made a sincere attempt was made to cover every bit of the data while coding.

The process of open coding in WEFT produced a total of 319 open codes across seven interviews.

**Fig. 1:**
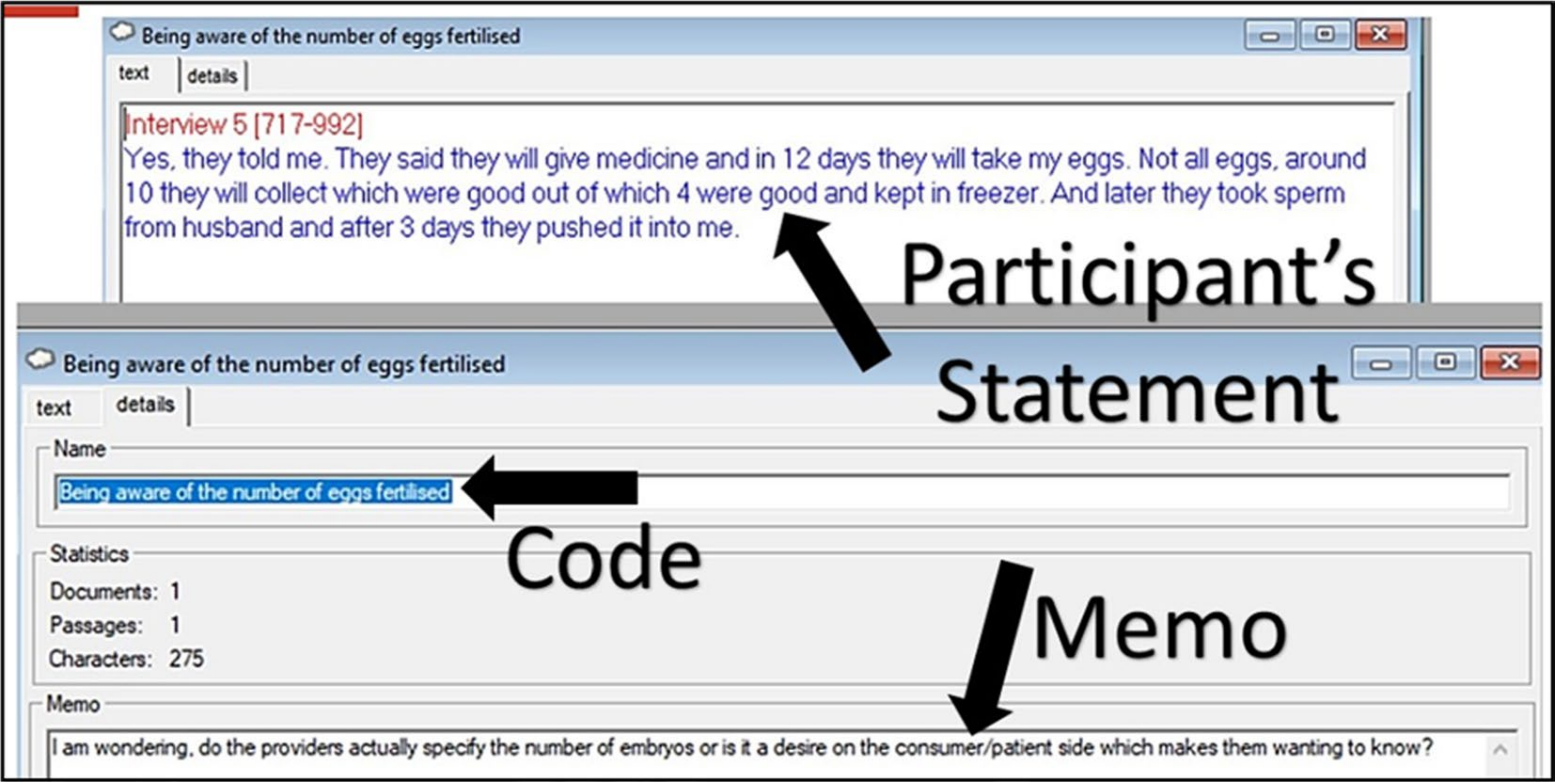
A typical open code in WEFT

This is how a typical code at the stage of open coding looked like in WEFT- Fig. 1.

(ii) Axial coding: The open codes were related to each other to form explanations via categories (axial codes). To make a large number of open codes easy to handle these codes were initially sorted into nine broad categories—(1) views of embryos, (2) reasons/motivations for IVF/IVF centre, (3) factors motivating/affecting decisions, (4) concerns/fears/inhibitions, (5) constraints/restrictions/mandatory procedure, (6) unaware/lack of awareness, (7) openness and satisfaction, (8) ambition/desire and (9) miscellaneous. These categories served as a precursor to axial codes.

Based on the categories, the open codes were further grouped using coloured bookmarks and chart papers to create axial codes.

The purpose of using coloured bookmarks and chart paper was to have a visual frame that would help in arriving at the axial codes. Once the axial codes were created all the open codes were rechecked to ensure nothing was ignored. At this point, the open codes which did not fit anywhere were classified as “Miscellaneous.”

The axial codes were further refined by merging similar open codes.

Finally, using Microsoft PowerPoint, a refined axial code with all its associated open codes and its strength was produced.

(iii) Selective coding: The “core axial code” was selected and related to all other codes producing a tentative GT (Corbin and Strauss 2014) (Noble and Mitchell 2016).

## Results

Interview time/setting/nature: Six interviews were held in person and one over the telephone. The average interview time was 32 min and 42 s with the shortest being 15 min and the longest being 50 min. Six interviews were held in English and one in Kannada with the help of an interpreter. All interviews except one were audio recorded. Handwritten notes were taken and later transcribed for the interview in which the participant refused to be audio recorded (Table 1).

**Table 1:** Demographics of the participants

Total number of participants	7
Age range	30 years – 53 years
Education	All participants had a university degree
Gender	6 Women and 1 Man
Employment	All participants had a full-time job at the time of the interview
Religion	4 Hindu and 3 Christian
IVF outcome	All participants had one or two children through IVF except for one who conceived naturally after a failed IVF attempt
Use of donor Gametes	Not reported by anyone
Infertility	Male factor, Female factor, Unknown factor, and a combination of two, all were reported
Language	English and Kannada
Profession	All employed full-time (including three medical professionals and one pharmacologist)

The axial codes, in relation to the objective of this study yield the following explanation.

### Knowledge of Spare Embryos

The desire for a biological child was present amongst all participants. This was often coupled with social and family pressure to conform to parenthood which had a bearing upon the way in which the participants would start their IVF journey and in their interest in wanting to know things beforehand. For example, one participant who is a medical professional said:

Because when I started with the process, though I am a doctor, I did not read anything about it (IVF) because I wanted to go fresh I didn’t want to have any preformed ideas about it because I didn’t start willingly, it was a pressure that it is not happening, family pressure about it that why it is not happening? Why you guys are doing nothing about it? Why are you not evaluating? So when I started I did not read about it I was actually not aware that how many embryos can be produced and how they’ll be stored. I was completely unaware of the entire process. (P3)

Participants did not always learn about spare embryos through IVF providers. Their own reflection and curiosity about the IVF process could add to their knowledge:

I exactly don’t remember what time it was, before the egg retrieval during that consent we were talking about it that is when I realized in case we had more. What to do with those? And what will happen to them? Like do I want later I want a child can they be kept for that? How long they are kept? That is when I thought about it when the egg retrieval time came. (P3)

Other participants learned about spare embryos from various sources. These included magazines, scientific journals, newspapers, another friend or colleague, a priest, a counsellor at the IVF center, or the gynecologist:

The … gynecologist has himself told, “the number of … depending on the number of follicles, after the retrieval you might have extra embryos” they have the maximum limit of four to be transferred so if at all any remaining they will go for cryopreservation or so. They have clearly explained it. (P1)

As you know my profession, I know about it (spare embryos), otherwise also! But yes where I underwent (IVF) they talked to me about it (Spare embryos). (P2)

All the participants knew about the number of embryos at one stage or another. However, a majority of them did not know about the various fates which spare embryos can be destined for. Only one participant admitted being thoroughly informed on the embryo donation option.

Interviewer: And you know that some people give their surplus embryos to research or to another couple are you that option some people take, you aware of that?

P6: That and all they (IVF provider) have not said!

A desire for success dominated the minds of most of our participants throughout their IVF journey. This affected their ability to ask more questions from IVF providers during the IVF process—

I feel that our system is in such a fashion that if I am suspicious I am distressing myself. They are not transparent and me trying to find out more about it or worrying about it will put me in stress. So I didn’t think about these aspects. (P7)

The context of being preoccupied with success and desperation for biological children undermined the purpose and process of informed consent for some participants. Two participants admitted the possibility of overlooking the Informed Consent Documents (ICD).

Interviewer: Do you think you read the documents which were shared in the consent process thoroughly?

P3: No. I don’t think I read it. Both of us just … We might have overlooked it. I don’t know if that information (on spare embryos) is given to us in the consent. We might have overlooked it. Because we had not gone through it in detail. We just saw the procedure-related things and the success rate.

Interviewer: Was there a separate consent form for the embryos?

P2: I don’t remember because at that time we were not really reading into it. We only went into for the concept for IVF so went and I thought I will come back with a child so we had no issue looking into all that.

### Perceptions of Embryos

Most participants held well-defined views on embryos. No two participants described how they perceived their embryos similarly, yet all of them expressed a sense of possessiveness towards their embryos.

On asking about embryo donation, the participants’ views on their embryos were most apparent. For example, one participant said,

I mean our own flesh and blood away from us … not possible. So finally we thought about research … But then again research we started having all kinds of negative thoughts so finally, we have to discard it. (P7)

The thought of leaving my embryo there, while my relation with my husband still on we gotta. We are perfect couples! I would have gone back for the second cycle. (P2)

Another participant says

I would have not given it for research and also not to another person as it will be a constant reminder (of struggle) in the future. (P4)

Some participants who desperately waited for a child perceived their children after a successful IVF as a reward. This made them more willing to donate spare embryos as according to them they already got their “reward.” Only one participant in our study was willing to give all her spare embryos to both research and another couple without expressing any interest in knowing about the recipient,

P5: I am willing (to donate embryos) because I got it so let others also get it.

Interviewer: Who is the other person getting the embryo would you want to know that?

P5: No I don’t want any details.

Interviewer: Why don’t you want it?

P5: I don’t want to know because I am happy with what I got. After eleven years I got the baby. So wherever they (recipients) are let them be happy.

Interviewer: You would also be happy to give it for research?

P5: I don’t have any problem.

Our participants generally expressed a positive view of research. They knew that research (including research on embryos) could have a positive impact on the lives of many. For example, a woman who categorically refused to donate her spare embryos for any kind of research also acknowledged the possibility of the benefits which may arise from research.

Long run it (donating embryos to research) may be useful for the scientific or medical field but I cannot accept a part of me being butchered or in whatever way used for something. I would have felt really guilty for doing that. (P3)

Another participant who also refused to donate to research acknowledges the benefits while making an interesting analogy—

Research and finding a solution for a disease is good. But I don’t know how I will feel about giving my progeny for research and that it (progeny) doesn’t have a say in it. If I am asked to give, I mean I was asked to give my liver biopsy or my blood for some investigations, I was ready for it. I had jaundice twice and they were saying if they can find out something with it. Ahh that I had no problem with, somebody will benefit so it’s okay probably that will benefit me only. But the thought of giving my progeny without them having a say in it … I may not have agreed on it. (P2)

It is interesting to note how she used the words “(progeny) doesn't have a say in it” to refuse embryo donation for research. It is suggesting that she believes that embryos have an autonomy of their own which cannot be sidelined.

Another area where perceptions about embryos clearly emerged was getting a chance to see them through an electron microscope. At least two participants in our study got this opportunity. Both remembered feeling joyful at the sight of embryos. One described it as a “mucous-like cells” the other as “my children at the four-cell stage.”

Yet another area in which participants very distinctly described their views on embryos is when they were directly asked about the controversy surrounding human embryonic stem cell research. One participant quite explicitly expressed his views in philosophical terms—

I would say that your awareness, your awareness of the self is an important part of being human. A cell itself having that thing, I am not very sure if it is there (with the embryos). I don’t know if I am right? (P7)

On the whole, even those participants who did not seem to believe in embryos having any moral status indicated a sense of ownership towards the embryos some even used the words like “selfish” and “possessive” in describing their views.

### Desiring More Psychological Support, Time, and Information

Limited success rates of IVF, age-related concerns, procedure-related concerns like ovarian hyper-stimulation, ablation, abortion, embryos, were all adding to the physical, mental and financial stress for the couple. This was expressed in their expectations from IVF providers as they reflect upon their experience—

I definitely feel that there should be a counsellor for preparing the couple throughout the process. And the feeling of having the embryos inside the lady which is like almost all the hormone for pregnancy are given as you feel like it and then suddenly … It is like a miscarriage. I was feeling it that you know losing the baby though it was not a baby it was just an embryo. So it feels like a miscarriage and if someone didn’t succeed with it they should still take care of the emotion. Because it was emotionally not taken care that well I decided not to go for it again. (P3)

Ahhh … maybe because I am also busy with work so who would I go and meet, who would I get the information from. Who would be able to convince me that research would be okay? Otherwise, I was always research-oriented. I thought that I would go for research but things change later on. (P7)

Financially it should be better. Mentally also because the success rate is not 100 per cent and they may get depressed when not successful. (P6)

**Fig. 2:**
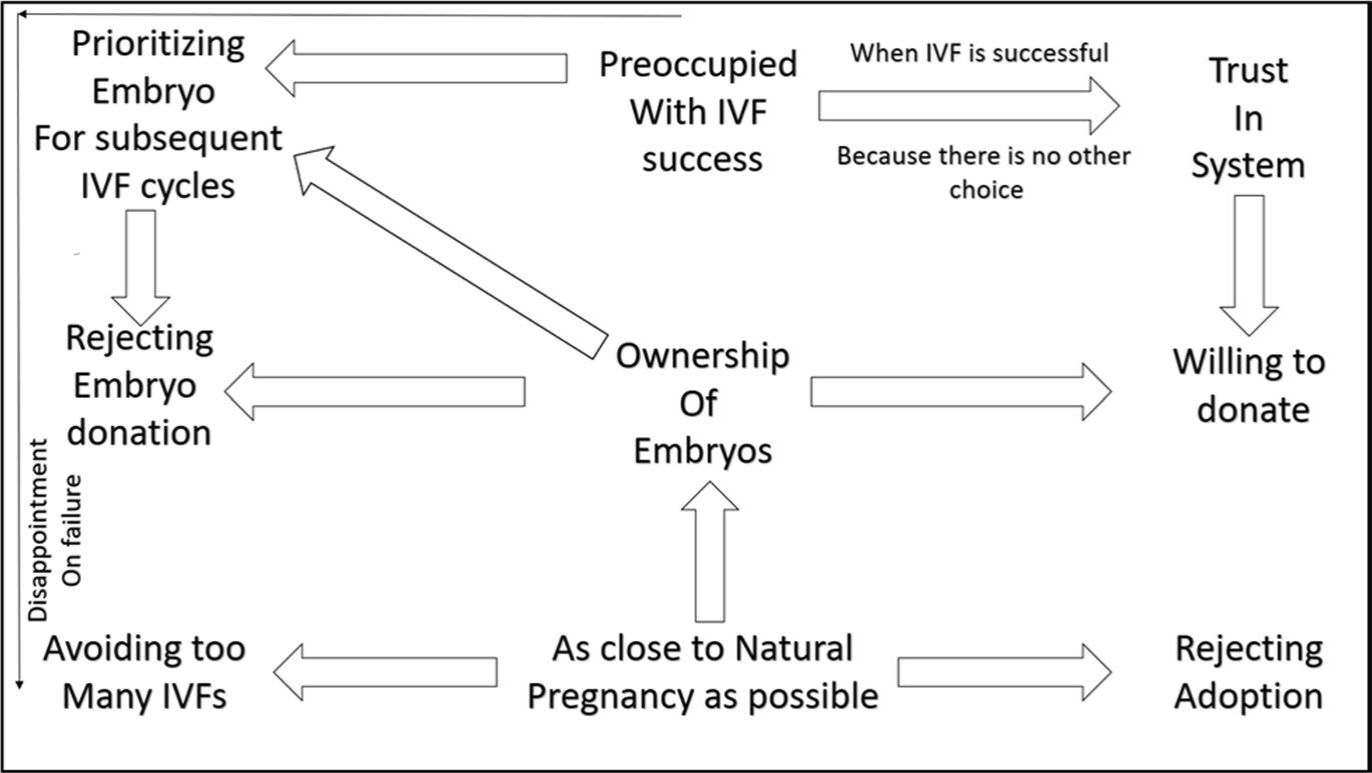
The relation between all the axial codes

With regards to the relationship between the axial codes, our study found that the ownership of the embryos is at the heart of it (Fig. 2).

A sense of ownership would determine participants’ decision to donate, reject donating embryos or prioritize embryos for the next IVF cycles. A desire to be as close to the natural pregnancy as possible led to a preference for a biological child (via IVF) and thus a rejection of options like adoption. Paradoxically, the “perceived unnaturalness” of the IVF manifested in experiencing stress (financial, physical, and emotional), less likelihood of success, and physical risks led to avoiding too many IVFs.

Cost friendly? No! As of now we have twins. We would have not gone for the second IVF. The whole pregnancy was very traumatic for my wife. Very bad. Every day in pain and a lot of issues. It was very stressful for me seeing her in pain and being a doctor not able to help her. So we decided, whatever was the result we would not go for another one. (P7)

The preoccupation with IVF success is yet another reason for prioritizing the spare embryos for future IVF cycles. And this also relates to trust in the ART providers and the medical system when the goals of a successful IVF are achieved. One of our participants mentioned that sometimes one has to trust because there is no other choice.

I feel that our system is in such a fashion that if I am suspicious I am distressing myself. They are not transparent and me trying to find out more about it or worrying about it will put me in stress. So I didn’t think about these aspects. (P7)

## Discussion

The first objective of this study was to assess the knowledge of IVF users on the status and outcome of their spare embryos. Here we found that all participants were generally able to recollect the number of embryos they had at one point or another during their IVF journey and a majority of them knew the outcome of their spare embryos. In this regard, our findings are a bit different from Gupta who interviewed twenty-eight women/couples attending IVF clinics in New Delhi and Mumbai during the IVF process and found that they often have limited awareness about surplus eggs/embryos (Gupta 2011). This the author mentioned was due to factors like cost, limited success rate, and other IVF-associated stress which makes them give little attention if any to the spare embryos. This difference could be because we interviewed participants after their IVF process was complete rather than during the process. They may have been better able to reflect upon the process and its outcome in retrospect. Interestingly, we found that participants may consciously choose to avoid researching/knowing about the IVF process beforehand to refrain from having any preconceived notions. Paradoxically, due to curiosity about the process of IVF, some IVF users may do extensive research which in addition to increasing knowledge can also add to the fear of a mishap with regard to embryos.

Though our participants could recollect having (or not having) spare embryos, many of them demonstrated poor recollection of the various disposal options. Chandy et al. in their study of eighty-seven IVF users also found that around one-third of them did not know about all of the embryo disposal options (Chandy et al. 2019). We also found that even the participants who recall having spare embryos could not recollect being given a choice in decision-making. This is likely because the clinic did not inform them about these options as evident from our interviews. The problem of inadequate or invalid informed consent in the context of IVF has been noted in other parts of the world as well. For example, Sahiner and Sehiralti (2023) have reported that a valid informed consent is not always a reality for people undergoing IVF in Turkey. Taking consent after starting the IVF procedure or sometimes even towards the end of the procedure was present. Consent procedures were also agnostic of the educational level of the IVF users. In Nigeria, too, lack of adequate informed consent despite there being guidelines has been described in the literature (Ajagunna 2023). An overall decrease in the quality of disclosure elements necessary for informed consent was noted by Krahn and Baylis (2016) in their review and analysis of Canadian IVF consent forms across several clinics from 1991 to 2014. However, just knowing about the prospects of spare embryos through informed consent may not be sufficient. “When” one learns about this prospect may also matter. A bit of appropriate information on the possibility of spare embryos and their various disposal options could change the client’s mind well before undergoing IVF. This is also discussed by Biggers and Summers in their paper titled “When to avoid creating surplus human embryos.” If the beliefs of the patients don’t allow them the destruction of any embryos and if they insist on IVF (i.e. refuse adoption) then options like limited ovarian stimulation, limited fertilization of eggs, and natural cycle IVF should be made available to them. It must, however, be explained to IVF users that these options may significantly lower their success rate (Biggers and Summers 2004). In India, the guidelines and the rules may not seem to take this point into consideration. For example, The National Guidelines for Accreditation, Supervision and Regulation of ART Clinics list several topics that need to be covered in counselling and the informed consent process. While it does talk about taking the patient’s consent before assigning the spare embryos to any particular fate it does not explicitly mention informing the clients about spare embryos from the very start of the IVF procedure (Indian Council of Medical Research 2005). Likewise, the recently passed ART Regulations Rules, 2022 provides a standard consent form template to be used before the start of the ART procedures but it too does not specify the possibilities of surplus embryos (Government of India, Assisted Reproductive Technology (Regulation) Rules, 2022).

A possibility of overlooking informed consent was expressed by some participants. This had two primary reasons as reported by participants themselves. The first was their own desperation for success and the second was the informed consent being too exhaustive. The stress and anxiety that accompanies IVF process can certainly add to this. These reasons were previously also noted in the literature by Gupta, Haimes, and Taylor, Madeira and Andraka-Christou, (Gupta 2011; Haimes and Taylor 2009; Madeira and Andraka-Christou 2016). Additionally, we also found that the most important aspects which were stressed during the IVF counselling procedure were “the success rate” and the “finances involved.” Unsurprisingly, as a result, every participant recalled these two pieces of information in our study. However, this could as well be because the participants themselves perceived these two factors as the most important piece of information for their IVF endeavours.

The concept of vulnerability is also important when it comes informed consent during IVF. People who undergo IVF can be “situationally vulnerable” for they are dependent on the IVF Providers for their treatment (Krahn and Baylis 2016). Additionally, during the initial phases of the IVF journey the patients can overestimate the prospects of IVF success and only subsequently can they have a better understanding of the oocytes (Carroll and Waldby 2012). These factors have implications on when should the IVF users be asked about their preferences for donating spare embryos. Carroll and Waldby suggest this should be after the first cycle is complete. In the Indian context too, Glasner and Gupta have argued that women in some senses are dependent upon a positive relationship with the IVF provider in the anticipation of a positive result. They also suggest that the paternalistic nature of the doctor–patient relationship in Indian societies will make the patients reluctant to inquire about their embryos (Glasner 2009; Gupta 2010). In our study, even though almost half of our participants were medical professionals themselves we captured a sense of “helplessness” in which one has to trust the system as there is no other choice. The vulnerability of the IVF patient could therefore be irrespective of the educational status which places more onus on IVF clinics to administer adequate informed consent including taking about spare embryos donation at the most appropriate time.

The second objective of this study was to explore the perceptions of IVF users of their spare embryos. Although no two participants described how they perceived their embryos similarly all participants indicated a sense of ownership towards their embryos. Embryos were described as a “reminder of struggle” as also reported by Nachtigall et al., a “symbol of a relationship with partner” as also mentioned in the findings of Jin et al. (Nachtigall et al. 2005; Jin et al. 2013), “progeny” as also discovered in the study by Kato and Sleboom-Faulkner and Provoost et al. (Kato and Sleboom-Faulkner 2011; Provoost et al. 2009). “Living entity” as also reported by Rosemann and Luo (2018), and one’s own flesh and blood. This characterization was independent of what our participants perceived as the moral status of the embryos—which showed diverse responses in our study. Elsewhere, there has been a common thread in respondents views on this subject. For example, in North America, Western Europe, and in Israel a common view is to perceive the successful implantation of an embryo as the start of a human life and not the embryos at pre-implantation stage (Kvernflaten et al. 2022; Roberts 2011; Raz et al. 2016). This view then translates into perceiving the implanted embryo as kin and the start of a potential relationship and familial responsibilities (Kvernflaten et al. 2022). There was no such trend in our study, which did have a small sample size. In addition to this, conceptualization of embryos as siblings to already existing children as mentioned in the works of Kato and Sleebom-Faulkner or a “potential life” as described in the research of Jin et al. also did not emerge in our studies (Kato and Sleebom-Faulkner 2011; Jin et al. 2013). One reason for not conceptualizing embryos as siblings to an existing child could be explained by the fact that a majority of participants in our study had undergone IVF for primary infertility and did not have any existing child as they underwent the process.

Our participants generally expressed a positive view towards research. They were supportive of all kinds of research that can benefit humanity. The positive views could be associated with the fact that many of our participants worked in a healthcare setting and had some experience of research themselves. Despite this, many categorically refused to donate their surplus embryos for research. Literature on this topic has highlighted that perception of science rather than embryos can influence IVF users’ willingness to donate for science (Provoost et al. 2010). However, several of our participants expressed a reservation to donate their embryos for research (or science) despite having favourable views of scientific endeavours. This area requires further inquiry. Samorinha et al. (2014) in their systematic review of thirty-nine studies (both qualitative and quantitative) on factors associated with embryo donation mentioned at least thirteen studies in which the conceptualization of embryos was directly associated with a reluctance to donate them for research. These studies were mainly from North America, Europe, and Japan. In our study, we observed that reasons for refusing to donate embryos for research is influenced by the moral perception of couples towards their embryos, adherence of religious beliefs and availability of information on the specifics of the research project.

Despite the overall positive views on research, one type of research that all participants unanimously opposed is enhancements and eugenics. Participants believed that even though they underwent IVF they would not like to engage in processes (eugenics) that they would have never otherwise had during a “natural pregnancy.” This theme of wanting to be as close to natural pregnancy as possible was also associated with having ownership of one’s embryos in addition to avoiding adoption and multiple IVFs since both these processes were perceived as “unnatural.”

The majority of our participants refused to donate their surplus embryos to another couple. The conceptualization of embryos and lack of information were two main reasons for the same. Studies by Chandy et al., Wanggren et al., and Raz et al. have found a greater preference for donating embryos to another couple as opposed to research amongst the IVF users in India, Sweden, and Israel respectively (Chandy et al., 2019; Wanggren et al. 2013; Raz et al. 2016). A common reason cited for the same in these studies is a desire to help other people going through the struggles of infertility. Complementarily, participants in our study would describe themselves as “selfish” and “possessive” while rejecting embryo donation to another couple. McMahon and Saunders and Newton et al. found that the ability to specify (or direct) the characteristics of the recipients could increase the will of couples to donate their embryos to another couple. The characteristics considered in their study included age, locality, religion, education level, and sexuality of the recipient among others (McMahon and Saunders 2009) and (Newton et al. 2007). In our study, the participants were generally determined in their decisions and besides more specific information on research projects and time, the only parameter which seemed to have a potential of changing their minds was when the recipient was someone they knew personally i.e., either a close friend or a family member. India’s family ethos is well reflected in this view. This could however also be due to trust and/or a sense of obligation towards the friend or family. Only one participant was willing to donate her surplus embryos to another couple and categorically refused donation to research. This was only because she perceived embryos as life and in so far as it is preserved, she would not have any objections. She did not express any desire to know about the recipients as long as she trusts the system.

In our study, given the small sample size, it is difficult to say whether the success of IVF, age, financial status, place of birth of participants, or the actual number of spare embryos had any considerable impact on the donation decision. We did, however, find that the professional profile of our participants had likely informed their perspectives on research involving embryos positively. However, it is important to note that the studies drawing the link between the demographics of the participants and their willingness to donate their surplus embryos are largely inconclusive (Samorinha et al. 2014).

### Limitations of the Study

All interviews were given either by solo woman/man and not by their respective spouses. Although one could say that in the absence of a spouse the participants expressed their individual opinions better, the lack of a spouse’s voice limits the scope to capture gender dynamics and collective decision-making within couples. Besides this, our participants were homogenous with regards to educational status (all had university degrees) and had an experience of working in healthcare settings. Thus, our findings may not be generalizable. With six women and one man, our study lacks the male perspective. The general limitations of a qualitative study like participants giving normative answers as opposed to what they actually thought/felt will hold true for our study as well. Finally, with seven participants alone, the study might not have reached information saturation nevertheless the findings do add to the discourse on this topic especially in the Indian context.

## Conclusion

We found that there is financial, social, economic, and psychological pressure on individuals in Dakshina Kannada who go through IVF. The process of decision-making on spare embryos occurs against the backdrop of these pressures. In the knowledge domain, the findings of our study reveal that the participants had a general awareness of the IVF procedure, the cost involved, the success rate and, the number of embryos they had at one stage of IVF or another. However, not all knew about the prospects of spare embryos from the very start of IVF. Many  of the participants demonstrated poor knowledge of the various fates that the spare embryos could have. Without the understanding and comprehension of various options, their decisions—whatever these might have been—cannot be considered informed. As for the perception of embryos, a majority of the participants expressed distinct views that were a product of their thought processes, beliefs, moral reasoning, and emotions. Even those who did not express any particular view asserted a sense of responsibility and ownership towards their embryos along with a general opposition towards embryo donation.

Overall, our study highlights the diversity of views on spare embryos from a selected Indian city and the need to strengthen informed consent procedures. An in-depth investigation on the way ICP unfolds in IVF clinics will help in formulating the best practices. What information is communicated to the IVF users in writing and what is told orally, when this information is communicated, how much of this information is retained, and which information may require repetition. Knowing each of these and more will help in determining the ways to uphold IVF users’ views and preferences with regards to their spare embryos. The diversity of views on embryos requires further empirical inquiry to establish if there is a common thread in India as it has been seen elsewhere. If ownership is the recurring theme, then this too requires further exploration. For example, is this ownership a joint one with the mother and father having an equal say or the one where either or the individuals or their families could have a bigger say? What happens in case of estrangement or divorce or the death of one person? While each of these topics have been discussed in Bioethics discourse (Asplund 2020) they need an exploration in the Indian context.

Given the population and the abundance of IVF clinics in the country the ethics of using spare embryos is the one which cannot be ignored.

## Data Availability

Authors are willing to share relevant anonymized data upon request.
